# An unsuspected complication with immune checkpoint blockade: a case report

**DOI:** 10.1186/s13256-018-1782-0

**Published:** 2018-09-04

**Authors:** Lucia Carril-Ajuria, Elisabeth Jiménez-Aguilar, Carlos Gómez-Martín, Carmen Díaz-Pedroche

**Affiliations:** 1 Medical Oncology Department, Hospital Universitario 12 de Octubre and Instituto de Investigación i+12, Madrid, Spain; 2 Internal Medicine Department, Hospital Universitario 12 de Octubre, Madrid, Spain

**Keywords:** Hypophysitis, Immune checkpoint blockade, Immune-related adverse events, Immunotherapy, Macroadenoma, Pituitary adenoma, Hyperthyroidism

## Abstract

**Background:**

Immunotherapy treatment with immune-checkpoint blockade has become a new paradigm in cancer treatment. Despite its efficacy, it has also given rise to a new class of adverse events, immune-related adverse events, which may affect any organ, including the thyroid and the pituitary.

**Case presentation:**

We present a case of a 77-year-old Caucasian man with metastatic renal cell carcinoma on immunotherapy treatment who was admitted to our hospital with a severe persistent headache of sudden onset. He had been on corticosteroid therapy for 10 days for suspected immune-related thyroiditis. The patient had tachycardia and mild diarrhea, and his thyroid function tests were compatible with subclinical hyperthyroidism with a suppressed thyroid-stimulating hormone level of 0.01 μIU/ml (0.4–4.5), a raised free T4 level of 2.17 ng/dl (0.7–1.9), and a free T3 level of 4.66 pg/ml (2.27–5). Computed tomography and magnetic resonance imaging revealed an enlargement of the pituitary gland compatible with macroadenoma. In the face of a probable immune-related hypophysitis, high-dose corticosteroid treatment was started. A posterior hormonal evaluation revealed secondary hypothyroidism with a suppressed thyroid-stimulating hormone level of 0.11 μIU/ml (0.4–4.5) and low thyroid hormones, a normal free T4 level of 1.02 ng/dl (0.7–1.9), and a low free T3 level of 1.53 pg/ml (2.27–5). These new findings suggested central hypothyroidism possibly due to pituitary apoplexy as a complication of the macroadenoma. Therefore, levothyroxine substitution was started along with the previously started corticosteroid therapy. The patient’s headache and asthenia gradually resolved, and after a few days, he was released from the hospital with levothyroxine substitution and corticosteroid tapering.

**Conclusions:**

This case emphasizes the importance of the differential diagnosis when dealing with patients on immune checkpoint inhibitors because other non-immune-related events may present. Our patient was finally diagnosed with immune-related hyperthyroidism and a concurrent pituitary macroadenoma. This case also highlights the importance of a prompt start of corticosteroid therapy once immune-related adverse events such as hypophysitis are suspected, because otherwise the outcome would be fatal.

## Background

Pituitary adenomas (PAs) are the most common cause of sellar masses (SMs), meaning about 80–90% of all SMs [1–3]. They are more frequent in women and elderly people. PAs may be classified as functional or nonfunctional, although this classification has been augmented by a more comprehensive system based on IHC studies of transcription factors. The 2017 World Health Organization classification officially requires routine IHC testing of the anterior lobe hormones and additionally requires transcription factors, such as PIT1, SF-1, and TPIT [4]. According to this classification scheme, hormone-negative adenomas may be classified in four groups: SF1-positive gonadotroph adenoma, PIT1-positive adenoma, TPIT-positive corticotroph adenoma, and transcription factor-negative null cell adenoma. Among all PAs, the clinically nonfunctioning or clinically silent PAs account for 25–35% [5]. However, careful studies of nonfunctioning pituitary tumors reveal that most actually do produce pituitary hormones. The most common hormones produced by nonfunctioning tumors are follicle-stimulating hormone (FSH), luteinizing hormone (LH), and the alpha subunit of FSH and LH. There are several reasons why these tumors seem nonfunctioning. The first reason is that FSH and LH excess do not usually cause specific symptoms. The second reason is that tumors arising from gonadotroph cells often only produce subunits of the hormones, which are not biologically active [6]. The third reason is that the hormones are usually secreted into the blood in small amounts, and therefore blood levels may be normal. PAs can also be classified by size as microadenomas, less than 1 cm, or macroadenomas, larger than 1 cm. There is a broad spectrum of nonpituitary SMs that may mimic the clinical picture of a PA, symptomatically, hormonally, and radiographically, such as gliomas, meningiomas; metastatic tumors; vascular lesions; and granulomatous, infectious, or inflammatory processes [3].

Hypophysitis is one of the nonpituitary SMs to be considered in the differential diagnosis. Inflammatory hypophysitis is a rare disease and can be classified into four different histological variants: lymphocytic, granulomatous, xanthomatous, and necrotizing. Lymphocytic hypophysitis, the most frequent, has an estimated annual incidence of 1 in 9 million people [7]. It can also be classified into primary hypophysitis; inflammation isolated to the pituitary gland of unknown origin; and secondary to medications, systemic diseases, infections, and so forth [8]. In secondary hypophysitis, there is a new entity, the immunotherapy-related hypophysitis, which, although it is a rare endocrine immune-related adverse event, could be fatal [9].

We present a case of immune-related hyperthyroidism and concurrent macroadenoma in a patient with a metastatic chromophobe renal cell carcinoma under immunotherapy treatment with an investigational anti-PD1 monoclonal antibody (mAb).

## Case presentation

A 77-year-old Caucasian man with metastatic chromophobe renal cell carcinoma under treatment with an anti-PD1 mAb was admitted to our hospital with a severe persistent occipital headache of sudden onset 12 hours before. The patient’s past medical history included hypertension, type 2 diabetes mellitus, and obstructive chronic bronchitis. He was a former smoker and had no drinking history. He used to work in finance and had no relevant family or environmental history.

The patient’s daily medications included antihypertensive medications, oral antidiabetics, omeprazole, and prednisone 25 mg daily. The headache spread to the front and both sides of the head and was associated with nausea and asthenia. It worsened with coughing and other valsalva maneuvers such as lying down. It did not get better with nonnarcotic pain killers, preventing the patient from falling asleep. However, the patient did not have diplopia, photophobia, phonophobia, or any other related symptoms. On admission, his blood pressure was 154/68 mmHg, his pulse was 101 beats/minute, his temperature was 36.7 °C, and his arterial blood oxygen saturation was 98%. The results of his physical and neurological examinations were otherwise unremarkable.

At the time of admission, the patient had been on corticosteroid therapy (0.5 mg/kg/d) for 10 days for suspected immune-related hyperthyroidism. He had tachycardia and mild diarrhea, and the results of his thyroid function tests were compatible with subclinical hyperthyroidism with a suppressed thyroid-stimulating hormone (TSH) level of 0.01 μIU/ml (0.4–4.5) with a raised free T4 of 2.17 ng/dl (0.7–1.9) and a free T3 of 4.66 pg/ml (2.27–5). The last dose of the anti-PD1 mAb, the 11th dose, had been administered 3 weeks before.

**Fig. 1:**
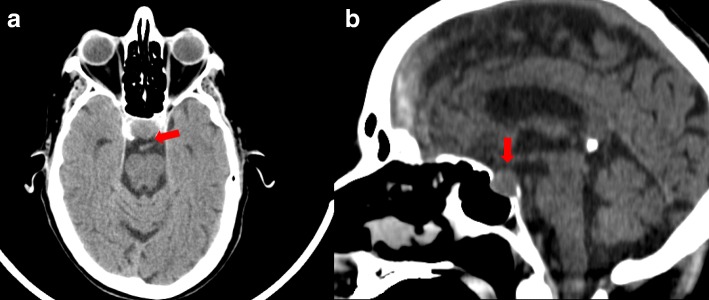
Arrows are pointing to the pituitary gland **a** and **b** Cranial computed tomographic scans showing an enlarged pituitary gland (15 × 20 × 14 mm) without calcification

**Fig. 2:**
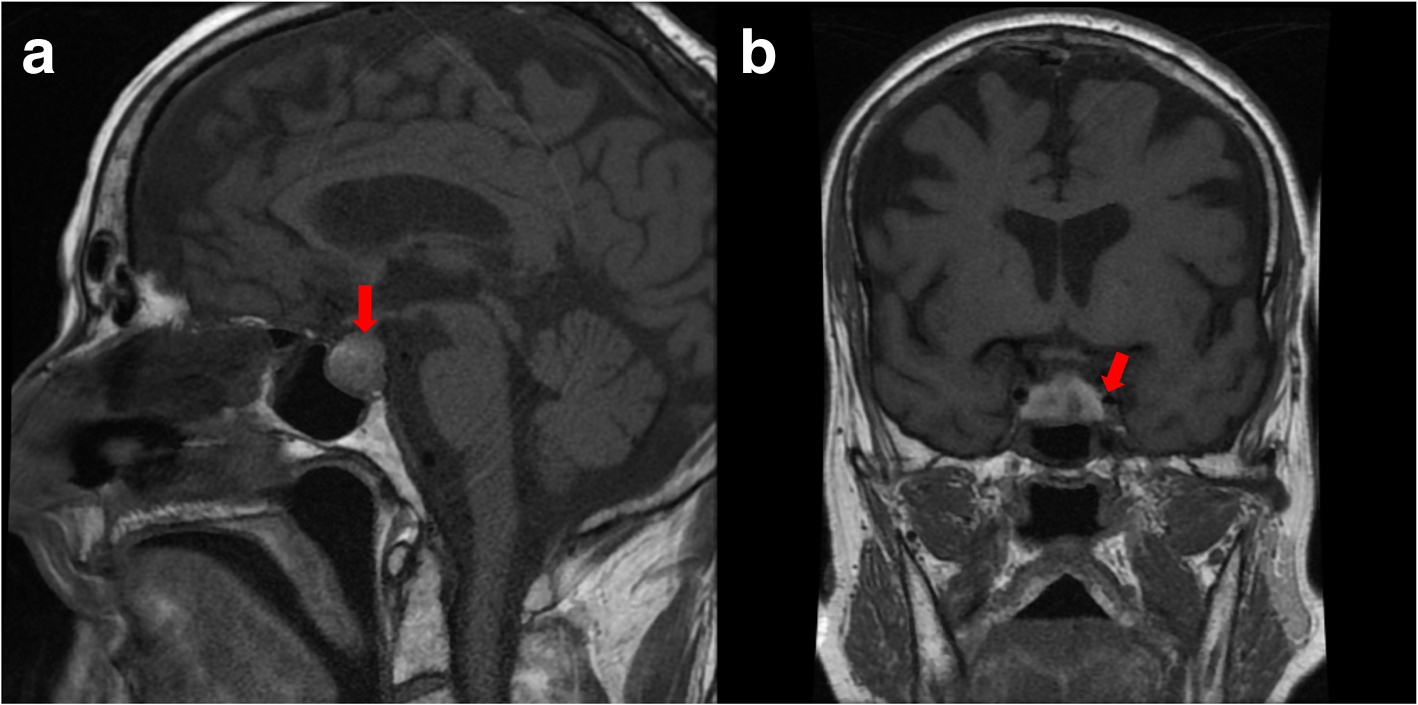
Arrows are pointing to the pituitary gland **a** and **b** Contrast-enhanced brain magnetic resonance imaging scans revealing a pituitary lesion with hemorrhagic areas enlarging the sella, compatible with a pituitary apoplexy

**Fig. 3:**
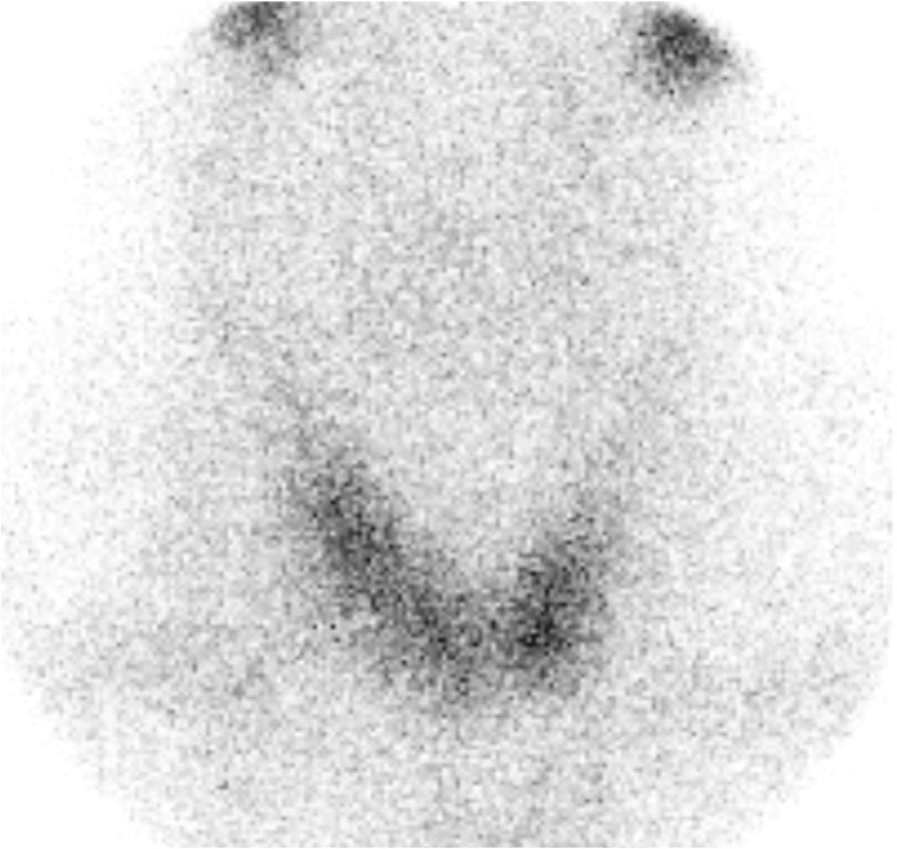
Thyroid scintigraphy showing irregular uptake throughout the thyroid gland

A cranial computed tomographic (CT) scan showed an enlarged pituitary gland (15 × 20 × 14 mm), compatible with macroadenoma, without calcifications (Fig. 1a and b). Taking into account the patient’s medical history, the initial diagnosis of an immune-related hypophysitis was assumed, and therefore we increased the corticosteroid dose to 1 mg/kg/d. Subsequently, a contrast-enhanced brain magnetic resonance imaging (MRI) scan revealed a pituitary lesion with hemorrhagic areas enlarging the sella, compatible with pituitary apoplexy (Fig. 2a and b). In addition, the patient had a thyroid disorder with a previous thyroid function test compatible with subclinical hyperthyroidism (TSH of 0.01 μIU/ml [0.4–4.5], free T4 of 2.17 ng/dl [0.7–1.9], and free T3 of 4.66 pg/ml [2.27–5]). The patient’s anti-thyroid peroxidase antibody, antithyroglobulin, and anti-thyroid-stimulating immunoglobulin antibodies were negative. Additional imaging studies were performed to clarify the cause of the primary hyperthyroidism. Ultrasound showed heterogeneous thyroid tissue with focal hypoechoic regions and irregular uptake on thyroid scintigraphy (Fig. 3). All of these findings, along with the patient’s palpitations and mild diarrhea, supported the hypothesis of immune-related subclinical hyperthyroidism in addition to a pituitary apoplexy. During the patient’s hospital admission, a hormonal reevaluation revealed a secondary hypothyroidism with TSH 0.11 μIU/ml (0.4–4.5), free T4 of 1.02 ng/dl (0.7–1.9), and free T3 of 1.53 pg/ml (2.27–5). Posterior evaluation of the hypothalamic-pituitary axis showed the following results: insulin-like growth factor 1, 136.1 ng/ml; growth hormone, 0.40 ng/ml; FSH, 8.73 mIU/L; LH, 6.18 mIU/L; testosterone, < 2.5 ng/dl; sex hormone-binding globulin, 3.50 mg/L, and prolactin, 5.2 ng/ml. These new findings suggested secondary hypothyroidism due to pituitary apoplexy.

Assuming the diagnosis of a primary immune-related hyperthyroidism, followed by a secondary hypothyroidism due to a macroadenoma complicated with a pituitary apoplexy, levothyroxine substitution was started along with the previous corticosteroid therapy. The patient gradually recovered from headache and asthenia and a few days later was released from the hospital with levothyroxine substitution (50 μg/d) and corticosteroid tapering. Six months later, aside from his chronic back pain, the patient remained asymptomatic. The whole-body CT scan carried out 1 week before the last visit showed a maintained partial response, and the last cerebral MRI showed a complete resolution of the hemorrhagic areas detected in the initial MRI. The results of the patient’s last thyroid function tests while receiving levothyroxine substitution (50 μg/d) were normal.

## Discussion

We report a case of a 77-year-old Caucasian with metastatic renal cell carcinoma on immunotherapy treatment who was diagnosed with a pituitary macroadenoma and concurrent immune-related hyperthyroidism. To our knowledge, this is the first reported case of pituitary macroadenoma and concurrent immune-related hyperthyroidism.

Immune-checkpoint inhibitors are an emerging therapy that has become a new paradigm for some types of cancer, such as melanoma, non-small-cell lung cancer, urothelial carcinoma, or renal cell carcinoma. Immune-checkpoint inhibitors are antibodies against specific immune-checkpoint molecules, such as cytotoxic T-lymphocyte-associated antigen 4 (CTLA-4) and programmed cell death protein (PD-1) and its ligand, that act by avoiding lymphocyte-mediated tumor cell destruction. By blocking these checkpoints, immune response against the tumor cells is upregulated. However, targeting these immune checkpoints may also cause self-tolerance dysfunction, which we refer to as immune-related adverse events (irAEs) [10].

irAEs have been described in almost every organ, including endocrine dysfunction. In a retrospective study conducted by Villa *et al.* [9], including patients who were treated with both anti-CTLA-4 (ipilimumab) and anti-PD-1 (nivolumab or pembrolizumab) therapies, endocrine dysfunction had a prevalence of 12.9% among all irAEs. Thyroid dysfunction was the most common endocrine irAE, being the most frequent subclinical hypothyroidism [9–11]. The spectrum of irAEs can differ, depending on the type of immune-checkpoint blockade. For instance, thyroid dysfunction has been described to have a higher incidence with anti-PD-1 therapies, whereas hypophysitis is more frequent after anti-CTLA-4 (10–15% with anti-CTLA-4 versus < 1% with anti-PD-1) [12–14].

The differential diagnosis of this case was difficult because both hypophysitis and PAs may present with a similar clinical picture with headache and fatigue, as our patient did. Luckily, these clinical symptoms along with the patient’s previous clinical history prompted us to request a pituitary MRI, which showed enlargement of the pituitary gland. In addition, a suppressed TSH and, later, low thyroid hormone levels suggested secondary hypothyroidism. Furthermore, although rare, the pituitary can be a site of metastasis from different cancers, including renal cell carcinoma, which conferred special difficulty in the differential diagnosis of our patient [15–19]. Therefore, all of these possibilities must always be considered in the differential diagnosis of a patient treated with immunotherapy who presents endocrine alteration and MRI pituitary enlargement. This case shows how relevant a proper differential diagnosis study is based on the previous medical history of the patient (headache, anti-PD-1 therapy, and a previous immune-related hyperthyroidism).

## Conclusions

This case shows the difficulty of the differential diagnosis when dealing with patients on immunotherapy. It also highlights the importance of the prompt start of corticosteroid therapy once an irAE such as immune-related hypophysitis is suspected, because otherwise the outcome could be fatal. Also, in addition to irAEs, it is important to take into account that other pathologies such as a pituitary macroadenoma may develop in oncologic patients treated with immunotherapy. It also emphasizes how critical a close thyroid function monitoring is when dealing with this type of drug. Thus, in our patient’s case, the secondary hypothyroidism that followed the initial primary hyperthyroidism was actually due to a pituitary macroadenoma and not to an immune-related hypophysitis.

## Abbreviations

CT: Computed tomographic
CTLA-4: Cytotoxic T-lymphocyte-associated antigen 4
FSH: Follicle-stimulating hormone
irAE: Immune-related adverse event
LH: Luteinizing hormone
mAb: Monoclonal antibody
MRI: Magnetic resonance imaging
PA: Pituitary adenoma
PD-1: Programmed cell death protein
SM: Sellar mass
TSH: Thyroid-stimulating hormone
